# Malignant peripheral nerve sheath tumor of the vagus nerve: an uncommon cause of progressive dyspnea

**DOI:** 10.1590/0100-3984.2016.0055

**Published:** 2017

**Authors:** Felipe Welter Langer, Daiane dos Santos, Giordano Rafael Tronco Alves, Gustavo Suertegaray, Carlos Jesus Pereira Haygert

**Affiliations:** 1 Department of Radiology and Imaging Diagnosis, University Hospital of Santa Maria, Federal University of Santa Maria (UFSM), Santa Maria, RS, Brazil

**Figure 1:**
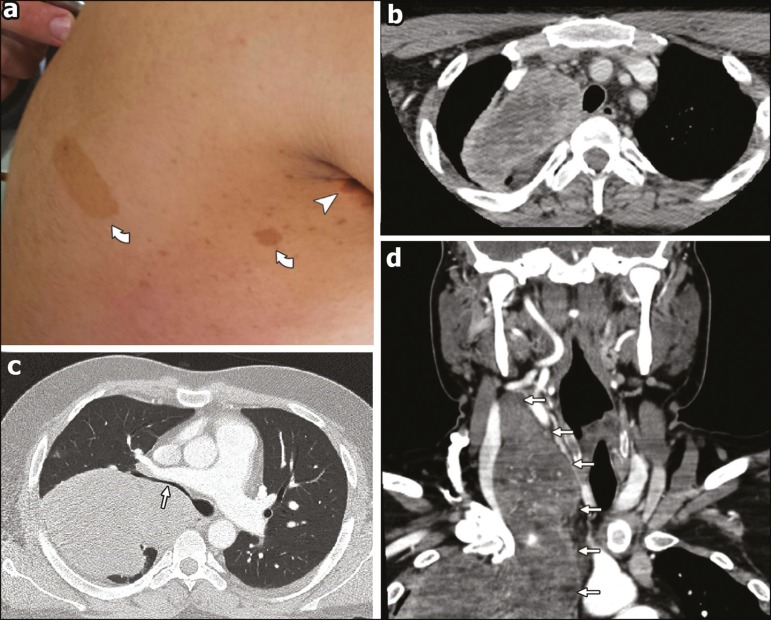
Findings on physical examination and CT. a: *Café-au-lait* spots (curved arrows) and axillary freckles (arrowhead) upon skin inspection. **b-d:** Axial CT scans of the neck (b,d) and chest (**c,d**) showing the MPNST. Note the heterogeneous enhancement after contrast administration (**b**) and the stenosis of the right main bronchus lumen (**c**), which accounts for the auscultation findings.

*Dear Editor*,

A healthy, nonsmoking, 27-year-old male patient was referred to our institution for investigation of a three-month history of progressive dyspnea. He reported that his dyspnea worsened on physical exertion and significantly limited his daily activities. He reported no cough, fever, night sweats, or weight loss; nor did he report any new lumps or masses during the last three months. Upon skin examination, multiple subcutaneous nodules and *café-au-lait* spots were noted, together with bilateral axillary freckles (Figure 1a). Collectively, those clinical findings met the criteria for a diagnosis of neurofibromatosis, which was so far undiagnosed. Pulmonary auscultation revealed diffuse wheezing in the right upper hemithorax. His biochemical profile was unremarkable. The patient then underwent a computed tomography (CT) scan of the chest with intravenous contrast administration, which revealed a 20-cm right cervicothoracic mass presumably arising from the right vagus nerve (Figures 1b-d). Because of the background of neurofibromatosis, a hypothesis of malignant peripheral nerve sheath tumor (MPNST) was raised and further confirmed by incisional biopsy and histological analysis. Given the proximity to vital structures, the patient was treated with a chemotherapy protocol for soft tissue sarcomas in an attempt to reduce the tumor bulk preoperatively. Because of a poor cellular response and recrudescence of the respiratory symptoms, the patient was deemed ineligible for any aggressive interventions.

MPNSTs are exceedingly rare sarcomas in the general population, with a lifetime risk of less than 0.01%. Conversely, in association with neurofibromatosis, these tumors arise in higher frequency because of malignant transformation from preexisting plexiform neurofibromas^(1)^. Overall, these tumors are associated with high local invasion, rapid growth, and early distant metastasis unless they are excised in a timely manner^(2)^. The most common locations for MPNST in neurofibromatosis patients are the extremities, head, and neck. Thoracic involvement, however, is remarkably rare, few cases having been reported^(3)^. According to the size and location of the intrathoracic tumor, compressive manifestations such as pain, dyspnea, dysphagia, and superior vena cava syndrome may be the presenting manifestations, as seen in our patient, who reported dyspnea as the sole symptom related to his MPNST^(3,4)^.

The identification of MPNST in neurofibromatosis patients may be troublesome for several reasons. First, the existence of multiple benign neurofibromas may delay the identification of changes in plexiform neurofibromas. In addition, because superficial cutaneous neurofibromas do not undergo malignant transformation, MPNSTs often remain undetected until they reach a moderate size or cause compressive symptoms. Furthermore, CT and magnetic resonance imaging might not be accurate enough to differentiate benign from malignant lesions with any degree of reliability in the very early stages, although advances have been made in the area of positron emission tomography^(4-6)^. Therefore, any suspicious lesions should generally prompt histological sampling^(7)^.

Although the mainstay of successful treatment of an MPNST is surgical excision after disease staging, neoadjuvant chemotherapy may be employed in order to reduce its dimensions beforehand, especially in patients with lesions surrounding vital organs. Radiotherapy might also delay recurrence, although it has not been shown to improve survival in MPNST patients^(8)^.
